# Myricetin Disturbs the Cell Wall Integrity and Increases the Membrane Permeability of *Candida albicans*

**DOI:** 10.4014/jmb.2110.10014

**Published:** 2021-10-30

**Authors:** Heung-Shick Lee, Younhee Kim

**Affiliations:** 1 Department of Biotechnology and Bioinformatics, Korea University, Sejongsi 30019, Republic of Korea; 2 Department of Korean Medicine, Semyung University, Jecheon 27136, Republic of Korea

**Keywords:** Antifungal, *Candida albicans*, crystal violet, leakage, membrane permeability, myricetin

## Abstract

The fungal cell wall and membrane are the principal targets of antifungals. Herein, we report that myricetin exerts antifungal activity against *Candida albicans* by damaging the cell wall integrity and notably enhancing the membrane permeability. In the presence of sorbitol, an osmotic protectant, the minimum inhibitory concentration (MIC) of myricetin against *C. albicans* increased from 20 to 40 and 80 μg/ml in 24 and 72 h, respectively, demonstrating that myricetin disturbs the cell wall integrity of *C. albicans*. Fluorescence microscopic images showed the presence of propidium iodidestained *C. albicans* cells, indicating the myricetin-induced initial damage of the cell membrane. The effects of myricetin on the membrane permeability of *C. albicans* cells were assessed using crystal violet-uptake and intracellular material-leakage assays. The percentage uptakes of crystal violet for myricetin-treated *C. albicans* cells at 1×, 2×, and 4× the MIC of myricetin were 36.5, 60.6, and 79.4%, respectively, while those for DMSO-treated *C. albicans* cells were 28.2, 28.9, and 29.7%, respectively. Additionally, myricetin-treated *C. albicans* cells showed notable DNA and protein leakage, compared with the DMSO-treated controls. Furthermore, treatment of *C. albicans* cells with 1× the MIC of myricetin showed a 17.2 and 28.0% reduction in the binding of the lipophilic probes diphenylhexatriene and Nile red, respectively, indicating that myricetin alters the lipid components or order in the *C. albicans* cell membrane, leading to increased membrane permeability. Therefore, these data will provide insights into the pharmacological worth of myricetin as a prospective antifungal for treating *C. albicans* infections.

## Introduction

*Candida albicans* is the leading fungal species causing nosocomial infections [1]; however, a noticeable shift towards non-*albicans* species of *Candida*, including *C. tropicalis*, followed by *C. glabrata* and *C. krusei*, has been reported [2]. *C. albicans* is a commensal that is present in 15–60% of symptomless individuals in the yeast form and commonly demonstrates yeast-to-hypha transitions along with tissue infiltration and infection [3]. In recent times, the incidence of lethal systemic candidiasis has increased dramatically on account of the increase in the numbers of seriously ill or immunocompromised patients, such as transplant recipients, patients with cancer and HIV infections, and patients showing the frequent use of invasive medical treatment [4]. Virulence factors of *C. albicans* contribute towards the pathogenesis of infections caused by this fungus; such virulence factors include adhesin molecules for host recognition, ability of morphogenesis between unicellular yeast cells and filamentous forms, and secretion of aspartyl proteases and phospholipases [5]. Additionally, the biofilm formation ability of *Candida* spp. is known to be implicated in the increased resistance to antifungal therapies and the host immune system [6].

The main targets of available antifungal drugs for the treatment of systemic fungal infections can be categorized as follows: the fungal cell membrane, cell wall, and nucleic acids. Inhibition of lanosterol 14α-demethylase by azoles causes the exhaustion of ergosterol, an analog of cholesterol, in the fungal cell membrane, and accumulation of sterol precursors, leading to alterations in the structure and function of the plasma membrane [7, 8]. Polyenes such as amphotericin B bind to and extract ergosterol directly, resulting in the disruption of many components of the yeast cell physiology [9, 10]. Furthermore, the interaction of amphotericin B with sterol causes membrane permeabilization via the production of ion channel formation, leading to the leakage of K^+^ and Na^+^ and the death of yeast cells [10]. Echinocandins are inhibitors of (1,3)-β-glucan synthase, a large integral membrane heterodimeric protein in the biosynthetic pathway of (1,3)-β-glucan, which is a major component of the fungal cell wall [11, 12]. Nucleotide analogs, including 5-fluorocytosine, inhibit nucleic acid synthesis [13].

Although extensive studies have been performed to explore new antifungal drugs, commonly available drugs for the treatment of candidiasis are rather limited due to the eukaryotic nature of fungi and the emergence of drug-resistant yeast strains resulting from the common and prolonged use of antifungals [14, 15]. Therefore, it is essential to explore novel antifungals that are both safe to use and effective against *Candida* infections. There has been a long history of people using natural products to cure diseases and ailments. The advantages of plant-derived products used in food or traditional medicine include their effectiveness against microbes or insects and relatively high safety, given their repeated use or application for centuries. Myricetin is a common plant-derived product; it is a member of the flavonoid class that is found commonly in berries, red wines, teas, and vegetables [16]. Myricetin is produced by plants from a variety of families, including *Myricaceae* [17], *Anacardiaceae* [18], *Polygonaceae* [19], and *Primulaceae* [20]; it possesses many pharmacological activities, including antioxidant [21], anticancer [22], antidiabetic [16], anti-inflammatory [23], analgesic [24], antifungal [25], and hepatoprotective properties [26]. Moreover, myricetin has been used to preserve foods containing oils and fats due to its ability to protect lipids against oxidation [16].

The present study illustrates that myricetin has antifungal activity against pathologically important *Candida* species including *C. albicans*, *C. glabrata*, *C. krusei*, and *C. parapsilosis*. The mechanism underlying the antifungal action of myricetin was evaluated against *C. albicans*, a major fungal pathogen, with a focus on cell wall integrity using the sorbitol protection assay, and cell membrane permeability using propidum iodide(PI)-staining, crystal violet uptake, intracellular material leakage, diphenylhexatriene (DPH)-binding, and Nile red-binding analyses.

## Materials and Methods

### Candida Strains and Growth Conditions

The *C. albicans* SC5314 (ATCC MYA-2876) and *C. parapsilosis* ATCC 22019 were purchased from the American Type Culture Collection (ATCC, USA), and *C. glabrata* ATCC 2001 (KCCM 50044, CBS 138) and *C. krusei* ATCC 6258 (KCCM 11426) were procured from the Korean Culture Center of Microorganisms (KCCM, Korea), respectively. *C. albicans* SC5314 was used for routine analysis and other strains were included as controls. Routine cultures were carried at 35°C in yeast mold (YM) broth (Difco, USA).

### Reagents

Myricetin was obtained commercially from Tauto Biotech (China). DMSO (Dimethyl Sulfoxide), amphotericin B, Calcofluor White (CFW) M2R, PI, crystal violet, DPH and Nile red were purchased from Sigma (USA). Phosphate Buffered Saline (PBS, pH 7.4) was procured from Gibco (USA) and Qubit dsDNA BR kit was purchased from Invitrogen (USA). Bradford reagent was obtained from Bio-Rad (USA). In addition, myricetin (20 mg/ml) was dissolved in DMSO, aliquoted, and stored at -20°C in the dark until use. Crystal violet (0.1 mg/ml) was dissolved in water, filtered, and stored at -20°C. Nile red (1 mg/ml) was dissolved in acetone and stored at 4°C.

### Antifungal Susceptibility Testing

Antifungal susceptibility to myricetin was evaluated for each strain by the standard broth microdilution CLSI M27-A3 method [27], using resazurin as a cell growth indicator [28].

Briefly, the two-fold serial dilutions of myricetin or amphotericin B (100 μl) were added to the wells of a round-bottom 96-well microplate containing RPMI-1640 medium. The inoculum suspension (100 μl) containing 0.1 mg/ml resazurin was added to attain a final cell density of 1 × 10^3^–5 × 10^3^ cells/ml, and the plate was incubated at 35°C for 24 h. Colorimetric MIC end-points were considered as the lowest sample concentration at which the solution remained blue, or the first sample whose color changed from blue to purple [28]. DMSO, which was the solvent used for the preparation of myricetin solution, was included as a growth control; no growth-inhibitory effects of DMSO were detected up to the concentration of 1%. Amphotericin B was used as a positive control.

### Sorbitol Protection Assay

To ascertain whether myricetin affects the *C. albicans* cell wall structure, the sorbitol protection assay [29] was performed using modified CLSI M27-A3 protocol, with resazurin [30]. In a 96-well round bottom microplate, two-fold dilutions of myricetin and two-fold dilutions of myricetin with 0.8 M sorbitol were added in two separate rows. All the wells were inoculated with *C. albicans* cell suspensions, and the plate was incubated at 35°C. The MIC values were evaluated at the 24- and 72-h time points.

### Microscopic Analysis

To examine the effect of myricetin on *C. albicans* cells, the myricetin-treated cells were observed using a confocal laser scanning microscope (CLSM) and a fluorescence microscope, respectively. First, log-phase *C. albicans* cells 1 × 10^8^ cells /ml were grown in the presence of 20 μg/ml myricetin or 1 μg/ml amphotericin B in YM broth at 35°C with agitation at 200 rpm. The cells were harvested either at 2.5 or 4 h by centrifugation at 12,000 ×*g* for 1 min and stained with 10 μg/ml PI in PBS. The cells were observed using a CLSM. Secondly, *C. albicans* SC5314 cells (1 × 10^8^ cells) were grown in the presence of 2 μl of DMSO or 40 μg myricetin per ml of YM broth at 35°C with shaking at 200 rpm for 2.5 h. The cells were harvested by centrifugation at 12,000 ×*g* for 1 min and stained with 10 μg/ml PI and 0.01% CFW in PBS. Then, they were observed using a fluorescence microscope equipped with triple RGB filters or a bright-field microscope.

### Crystal Violet-Uptake Assay

To evaluate the effects of myricetin on membrane permeability, the crystal violet-uptake assay was performed according to the method described by Vaara and Vaara [31] with slight modifications. Log-phase *C. albicans* SC5314 cells were harvested by centrifugation at 12,000 ×*g* for 5 min, and then washed and resuspended in PBS. Cell suspensions (5 × 10^7^ cells/ml) were treated with 1×, 2×, or 4× the MIC of myricetin and incubated at 35°C with shaking at 200 rpm for 30 and 60 min. Solvent (DMSO) controls for each treatment of myricetin were included. Then, the cell suspensions (0.9 ml) were harvested by centrifugation at 12,000 ×*g* for 5 min and washed in PBS. The cells were suspended in 1 ml of PBS containing 10 μg/ml crystal violet and incubated at 35°C with shaking at 200 rpm for 15 min. Further, the cells were precipitated by centrifugation at 12,000 ×*g* and 4°C for 20 min, and the supernatant (0.2 ml) was placed in quadruplicate into a 96-well flat-bottom microplate. The amount of crystal violet remaining in the supernatant was measured as the absorbance at 590 nm (A_590_) using a spectrofluorometer (Tecan, Austria). The optical density values of the initial solution of crystal violet used in the assay were regarded as 100%. The percentage of crystal violet uptake was calculated using the following formula: uptake of crystal violet (%) = 1-A_590_ of the sample/A_590_ of crystal violet solution × 100.

### Leakage of Intracellular Materials

An evaluation of myricetin-induced nucleotide and protein leakage was performed using a fluorometric and spectrophotometric method, respectively [32]. An overnight culture of *C. albicans* SC5314 cells was diluted by 1:5 into fresh YM broth and incubated further at 35°C with shaking at 200 rpm for 3 h. The cells were harvested by centrifugation at 12,000 ×*g* for 5 min, washed with PBS, and resuspended in PBS to achieve a cell density of 1 × 10^8^ cells/ml. The cell suspensions were then incubated with 40 and 80 μg/ml myricetin at 35°C with agitation at 200 rpm for 30 or 60 min. DMSO controls for each myricetin treatment were included. The cell suspensions (0.8 ml) were centrifuged at 13,200 ×*g* at 4°C for 20 min, and the supernatants were saved for further analysis. For the nucleotide leakage analysis, the Qubit dsDNA BR assay kit and a Qubit 4 Fluorometer was used; this kit measures the levels of double-stranded DNA over RNA highly selectively. The supernatant (20 μl) were mixed with 180 μl of working solution in triplicate and the fluorescence of these mixtures was measured. The concentrations of the nucleotides in the samples were calculated using the dilution calculator feature of the Qubit 4 fluorometer. For protein leakage analysis, the Bradford assay [33] was performed according to the manufacturer’s instructions. Diluted Bradford concentrate (150 μl) mixed with 50 μl of supernatant or PBS was added to a 96-well clear flat-bottom microplate in quadruplicate, and the absorbance of the samples at 590 nm was measured using a spectrofluorometer. The amount of protein leakage was calculated as the A_590_ of the sample − the A_590_ of Bradford solution containing PBS.

### DPH-Binding Assay

To monitor whether myricetin affects the lipid components or order in the *C. albicans* cell membrane, the DPH-binding assay was performed [32]. *C. albicans* SC5314 cells (1 × 10^8^ cells/ml) at the log phase were incubated with myricetin or DMSO at 35°C with shaking at 200 rpm for 30 min. The cells (0.9 ml) were then harvested by centrifugation at 12,000 ×*g* for 5 min, washed with PBS, and resuspended in 0.9 ml of PBS containing 50 μM DPH. The cell suspension (0.2 ml) was then transferred in quadruplicate to a 96-well black flat-bottom microplate, followed by incubation for 10 min in the dark at room temperature. The amount of DPH binding to the *C. albicans* cell membrane was measured using a spectrofluorometer (Tecan, Austria) at 360 nm (bandwidth, 35 nm) and 460 nm (bandwidth, 10 nm) as the excitation and emission wavelengths, respectively. The DPH-binding percentage was calculated using the following formula: relative DPH binding (%) = (F_myricetin_ – F_PBS containing DPH_)/(F_DMSO control_ – F_PBS containing DPH_) × 100; F represents the fluorescence intensity.

### Nile Red-Binding Assay

The Nile red-binding assay was performed as follows: *C. albicans* SC5314 cells in the exponential growth phase were harvested by centrifugation at 12,000 ×*g* and the precipitate was suspended in PBS. The cell suspension (2 × 10^7^ cells/ml) was exposed to myricetin (from 20 to 80 μg/ml) or an equivalent amount of DMSO at 35°C with agitation at 200 rpm for 1 h. Then, the cells (0.9 ml) were harvested by centrifugation at 12,000 ×*g* for 15 min, washed with PBS, and suspended in 0.9 ml of PBS containing 0.25 mg/ml Nile red solution. The cell suspension (0.2 ml) was transferred in quadruplicate to a 96-well black flat-bottom microplate, followed by incubation for 5 min in the dark at room temperature. The amount of Nile red binding to *C. albicans* cells was measured using a spectrofluorometer at 488 nm (with a bandwidth of 20 nm) and 580 nm (with a bandwidth of 20 nm) as the excitation and emission wavelengths, respectively. The Nile red-binding percentage was calculated using the following formula: relative Nile red binding (%) = (F_myricetin_ – F_PBS containing Nile red_)/(F_DMSO control_ – F_PBS containing Nile red_) × 100; F represents the fluorescence intensity.

### Statistical Analysis

All experiments were performed at least twice in triplicate or quadruplicate. For each outcome, the data were represented as mean ± standard deviation. The effect of myricetin compared with controls was analyzed using SigmaPlot 13.0. A *p* value less than 0.05 was regarded as statistically significant.

## Results and Discussion

### Antifungal Susceptibility Testing

Higher plants defend against pathogens with secondary metabolites or antimicrobial compounds including polyphenols, such as myricetin. The MIC of myricetin against *C. albicans* SC5314, a strain used for routine assays in several fungus-related studies, was 20 μg/ml. The MIC values of myricetin against *C. glabrata* ATCC 2001, *C. krusei* ATCC 6258, and *C. parapsilosis* ATCC 22019 were 1.3, 5, and 5 μg/ml, respectively (Table 1). In contrast, the MIC values of amphotericin B against the tested *Candida* species ranged from 0.5 to 1 μg/ml. Myricetin appears to have moderate anticandidal activity and the data agree reasonably with other researcher’s MIC values of 16-64 μg/ml against *C. albicans*, 3.9 μg/ml against *C. glabrata*, 64 μg/ml against *C. krusei*, and 54 μg/ml against *C. tropicalis* [25]. The reason why the MIC value of myricetin is considerably higher (20 μg/ml) than that of amphotericin B (1 μg/ml) against *C. albicans* SC5314 is due to their differences in cellular targets and structures, although both amphotericin B and myricetin induce increased membrane permeability to result in cell death.

### Sorbitol Protection Assay

The fungal cell wall surrounding cell membrane affords cells strength and rigidity and maintains osmotic support from the turgor pressure of protoplasts. Impairments in cell wall components by antifungals will result in cell lysis, but cells can survive in the presence of an appropriate osmotic protectant in the medium [29]. To examine whether the antifungal activity of myricetin is related to the alteration of the fungal cell wall structure, the sorbitol protection assay was performed using the CLSI M27-A3 microdilution assay with myricetin against *C. albicans* cells with or without 0.8 M sorbitol (Table 2). In the presence of sorbitol, the MIC values of myricetin against *C. albicans* increased from 20 to 40 and 80 μg/ml in 24 and 72 h, respectively. The increase in the MIC values in the sorbitol protection assay indicates that myricetin is involved in disrupting the integrity of the *C. albicans* cell wall.

### Microscopic Analysis

**Fig. 1:**
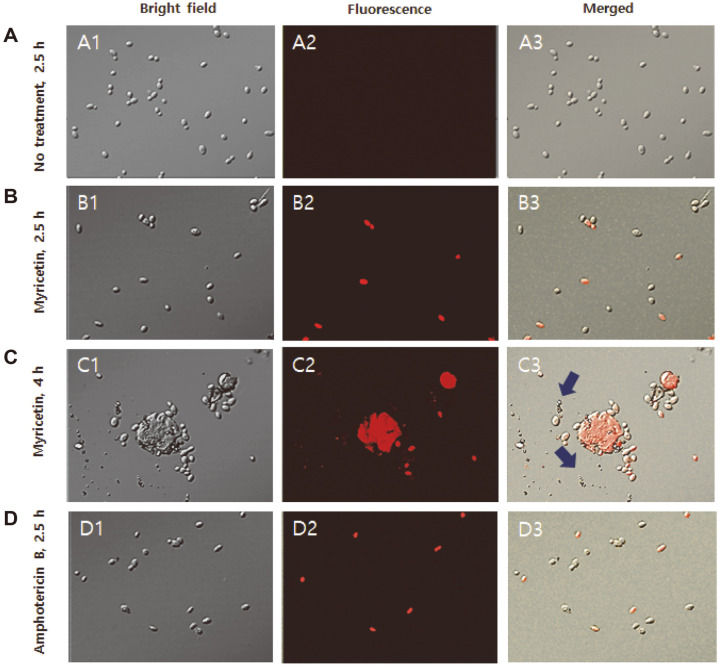
Confocal laser microscopic images of *C. albicans* SC5314 cells. *C. albicans* cells were incubated with DMSO (A1, A2, and A2) for 2.5 h, 20 μg/ml myricetin for 2.5 h (B1, B2, and B3) and 4 h (C1, C2, and C3), or 1 μg/ml amphotericin B for 2.5 h (D1, D2, and D3). The cells were stained with 10 μg/ml PI.

PI can bind to DNA and RNA through compromised cell membranes, but it is mostly eliminated from live cells. Therefore, PI can enter dead or dying cells with defective cell membranes and emit a red fluorescence signal, while live cells with intact cell membranes are not stained with PI [34]. As can be seen in Fig. 1B2, CLSM images show the presence of red PI-stained myricetin-treated *C. albicans* cells, suggesting that the *C. albicans* cells showed an initial impairment of the cell membrane after treatment with 20 μg/ml myricetin for 2.5 h. Although cell lysis was not detectable in the cells treated with myricetin for 2.5 h, they were noticeable after myricetin treatment for 4 h, as indicated by arrows in Fig. 1C3. Amphotericin B-treated *C. albicans* cells, the positive controls, were seen as fluorescent red cells with an intact form (cell wall), indicating that amphotericin B is involved in damaging the *C. albicans* cell membrane (Fig. 1D2). Amphotericin B, which is a polyene macrolide, is reported to participate in the formation of protein-like ion channels in the cell membrane [35,36] or the disruption of the polar head group region of biomembranes [37]. Lysed cell debris (Fig. 1C3), a characteristic of cells with a damaged cell wall, is easily found in myricetin-treated *C. albicans* cells. Thus, the results of the sorbitol protection assay and confocal laser microscopic analysis demonstrated that myricetin disrupts the cell wall integrity and injures the *C. albicans* cell membrane.

**Fig. 2:**
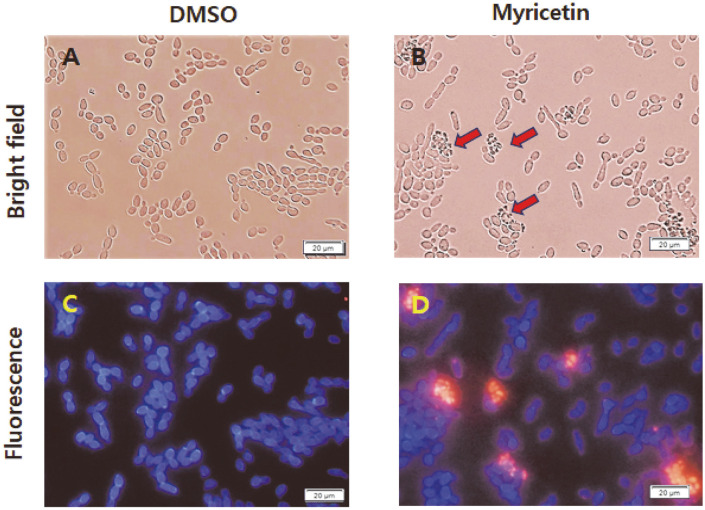
Microscopic images of *C. albicans* SC5314 cells. *C. albicans* cells were incubated with DMSO (**A** and **C**) or 40 μg/ml myricetin (**B** and **D**) and double-stained with 10 μg/ml PI and 0.01% CFW. Bright-field (**A** and **B**) and fluorescence (**C** and **D**) images of the cells are shown. The *C. albicans* cell walls were stained blue with CFW (**C** and **D**) and the cells with injured membranes were stained red with PI (**D**). Scale bars: 20 μm.

In addition, *C. albicans* cells treated with DMSO or 40 μg/ml myricetin for 2.5 h were stained with both CFW and PI. CFW is a fluorescent dye that stains fungal cell walls, which are composed of cellulose, chitin, and other β-1,4-carbohydrates [38]. As seen in Fig. 2C, the control *C. albicans* cells showed fluorescent blue cell walls stained with CFW, and no significant red fluorescence was detected, demonstrating that the cells had intact cell walls and membranes. In contrast, red fluorescent aggregates were found in case of myricetin-treated *C. albicans* cells (Fig. 2D). Furthermore, these PI-stained cells looked atrophied and formed cell aggregates, as indicated by red arrows in a bright-field image (Fig. 2B). These aggregates or clumps were generally detected when *C. albicans* cells were exposed to relatively high myricetin concentrations, such as 2× or 4× the MIC of myricetin, or sublethal concentrations of myricetin for a long time (> 4 h). We assume that large membranous clumps and cell aggregates may be formed by membrane fusion and ionic interactions between protoplasts, respectively, in case of myricetin-treated *C. albicans* cells because they have a compromised cell wall.

### Crystal Violet-Uptake Assay

Crystal violet or gentian violet exists as a lipophilic cation at neutral pHs. Although it does not penetrate cells with intact cell membranes, crystal violet enters cells with damaged cell membranes. Hence, the crystal violet-uptake assay is generally used for the detection of membrane impairment. As myricetin-treated *C. albicans* cells were seen as PI-stained fluorescent red cells, these were identified as membrane-damaged cells (Figs. 1 and 2). Therefore, the crystal violet-uptake assay was performed to ascertain whether myricetin affects the membrane permeability of *C. albicans* cells.

**Fig. 3:**
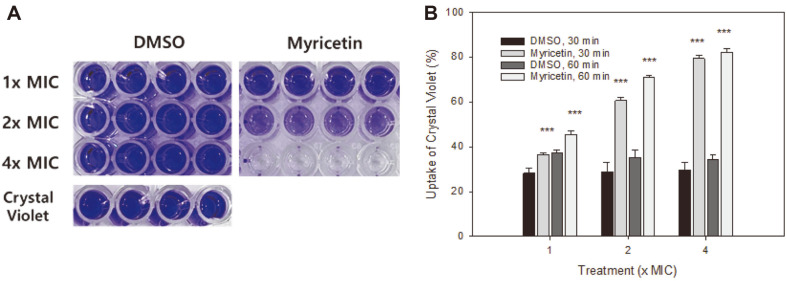
Crystal violet-uptake assay. (**A**) *C. albicans* cells treated with myricetin or DMSO for 30 min were harvested, suspended in crystal violet solution, and incubated further for 15 min. The cells were precipitated by centrifugation and the supernatant was placed into a 96-well flat-bottom microplate, as shown. (**B**) The amount of crystal violet in the supernatant was measured as the absorbance at 590 nm. The relative uptake of crystal violet (%) of myricetin- or DMSO-treated *C. albicans* cells is presented as the mean ± standard deviation. Representative data from one of three independent experiments is shown. ***p < 0.001: DMSO control vs myricetin-treated sample.

*C. albicans* cells treated with 1×, 2×, and 4× the MIC of myricetin or an equivalent amount of DMSO for 30 min were subjected to the crystal violet-uptake assay; the cell supernatants were placed in a 96-well microplate, as shown in Fig. 3A. There was a notable difference in color between the supernatants of cells treated with each concentration of myricetin supernatant and the supernatants of those treated with equivalent amounts of DMSO (control); this difference was concentration-dependent (Fig. 3A). The percentages of crystal violet uptake by *C. albicans* cells treated with 1×, 2×, and 4× the MIC of myricetin for 30 min were 36.5, 60.6, and 79.4%, while those by *C. albicans* cells treated with the corresponding amounts of DMSO were 28.2, 28.9, and 29.7%, respectively (Fig. 3B). The difference between each myricetin-treated sample and an equivalent DMSO control was statistically significant (*p* < 0.001), and these data clearly demonstrate that myricetin markedly increases the membrane permeability of *C. albicans* cells.

### Leakage of Intracellular Materials

**Fig. 4:**
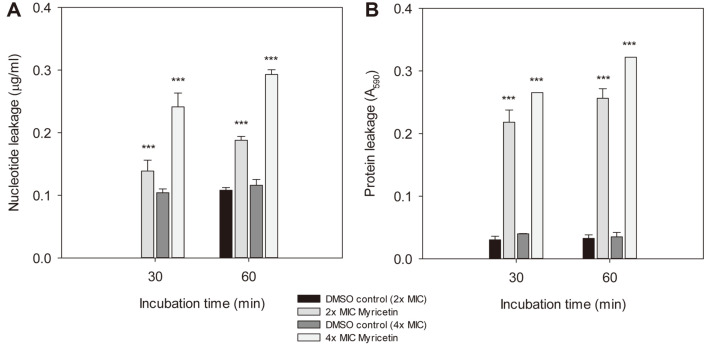
Nucleotide and protein leakage. *C. albicans* cells suspended in PBS were incubated with 2× or 4× the MIC of myricetin and centrifuged after the indicated time periods. Then, the supernatants were analyzed for nucleotide leakage (**A**) or for protein leakage (**B**). The data are shown as the means ± standard deviations. Representative data from one of three independent experiments are shown. ***p < 0.001: DMSO control vs myricetin-treated sample.

Since the notable enhancement of the membrane permeability in myricetin-treated *C. albicans* cells was displayed via the crystal violet-uptake assay, whether the treatment of *C. albicans* cells with myricetin causes the leakage of nucleotides and proteins was examined using the fluorometric and spectrophotometric method, respectively. *C. albicans* cells treated with DMSO or myricetin for 30 or 60 min were centrifuged and the supernatants were subjected to an analysis of the leakage of intracellular materials. As seen in Fig. 4A, DNA leakage levels of 0.139 and 0.241 μg/ml were found in case of the *C. albicans* cells treated with 2× and 4× the MIC of myricetin for 30 min, respectively, but a negligible amount and 0.104 μg/ml of DNA leakage were detected in the *C. albicans* cells treated with equivalent amounts of DMSO (0.2 and 0.4% DMSO), respectively. The difference observed between the DNA leakage levels in the myricetin-treated cells and the corresponding DMSO-treated controls was significant (*p* < 0.001).

For the protein leakage analysis, the A_590_ was measured after Bradford reagent was mixed with the supernatant (Fig. 4B). The absorbance values at 590 nm at 30 min were 0.030 and 0.040 in case of the control cells treated with 2× and 4× the MIC of DMSO, respectively, but were 0.218 and 0.281 in case of the *C. albicans* cells treated with 2× and 4× the MIC of myricetin, respectively. The difference between the A_590_ values of each myricetin-treated cell sample and the corresponding DMSO-treated control cell sample was also significant (*p* < 0.001). As revealed by the results of the crystal violet-uptake assay (Fig. 3) and the analysis of the leakage of intracellular materials (Fig. 4), the antifungal effects of myricetin against the membrane permeability of *C. albicans* cells were remarkable.

### The Binding of DPH into *C. albicans* Cell Membranes

To keep the viability of *C. albicans* cells, maintaining the integrity of the cell membrane is critical. Cells can regulate membrane function through regulating membrane fluidity and membrane protein arrangement. Therefore, changes in membrane permeability are related to alterations in membrane fluidity, which occur via changes in the lipid composition or order or pore formation. Hence, whether the increase of membrane permeability is associated with the lipid composition or order of *C. albicans* cell membrane was investigated using the DPH- or Nile red-binding assay.

**Fig. 5:**
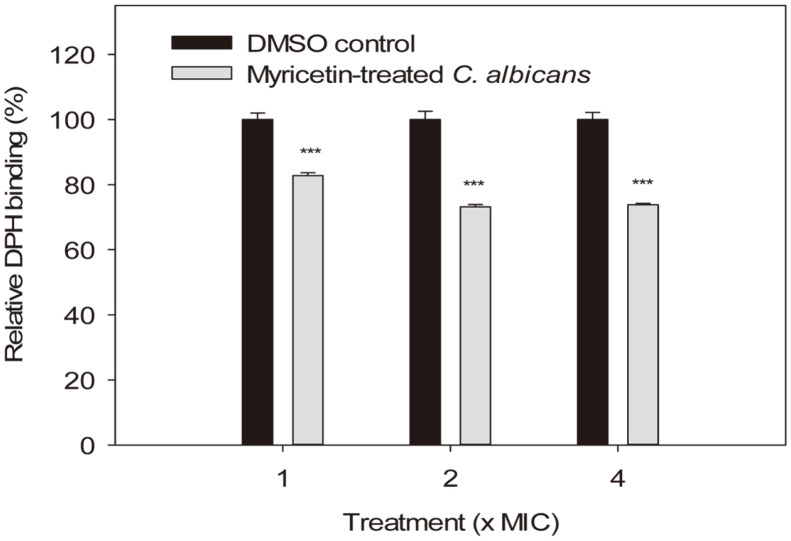
Entry of DPH into *C. albicans* cell membranes. Myricetin- or DMSO-treated *C. albicans* cells were incubated in PBS containing 50 μM DPH, and the fluorescence intensity of each sample was measured at 360 nm (bandwidth, 35 nm) and 460 nm (bandwidth, 10 nm) as the excitation and emission wavelengths, respectively. The data represent the means ± standard deviations obtained from one of three independent experiments. ***p < 0.001: DMSO control vs myricetin-treated sample.

DPH is almost non-fluorescent in water, but it shows a strong fluorescence after intercalation into membranes. Therefore, it can be used as a probe for viscosity, polarity, and lipid order [39]. DMSO- or myricetin-treated *C. albicans* cells were incubated with PBS containing DPH, and the fluorescence intensity of each sample was measured. The relative percentages of DPH binding to *C. albicans* cells treated with 1×, 2×, and 4× the MIC of myricetin for 30 min were 82.8 ± 0.9, 73.2 ± 0.7, and 73.8 ± 0.5%, respectively, compared to the corresponding DMSO controls (Fig. 5). The difference between each myricetin-treated cell sample and the corresponding DMSO-treated control cells was significant (*p* < 0.001), and there was no significant difference between the DPH binding (%) of the cells treated with 2× and 4× the MIC of myricetin. Consequently, decreased DPH entry into myricetin-treated *C. albicans* cell membranes suggest that myricetin causes alterations of the lipid components or order of *C. albicans* cell membranes.

### The Binding of Nile Red to *C. albicans* Cell Membranes

Nile red is a fluorescent probe that is used as a lipid stain to visualize bacterial cell membranes [40]. This dye displays low fluorescence in a polar environment, but selectively binds to lipids and emits strong fluorescence when incorporated into hydrophobic cell membranes [41, 42]. The penetration depth and the orientation of Nile red may vary in membranes with different lipid compositions; this could affect its fluorescence-emission ratios [43]. Therefore, Nile red can be used for monitoring the organization, fluctuation, and heterogeneity in membranes, specifically for membranes containing cholesterol [41, 44]. Accordingly, it was assumed that the binding of Nile red to cells would be changed if myricetin influences the lipid composition or organization of the cell membrane in *C. albicans* cells. As shown in Table 3, the relative binding of Nile red to myricetin-treated *C. albicans* cells was drastically reduced. After treatment with 20, 40, and 80 μg/ml myricetin for 1 h, the relative percentages of Nile red binding to *C. albicans* cells were 72.0, 64.6, and 55.7%, respectively, compared with the case for the corresponding DMSO-treated control cells. Consequently, a significant reduction of Nile red binding after myricetin treatment suggests that myricetin is induces alterations in the lipid composition or arrangement, such as the loosening of the packing of the lipid bilayer in cell membrane, leading to the enhanced membrane fluidity of *C. albicans* cells. Interestingly, treatment with 0.1 to 0.4% DMSO increased the fluorescence intensity of *C. albicans* cells (Table 3), since DMSO is a stain carrier that helps Nile red penetrate through the cell wall and cell membrane in microorganisms [45]. Nile red is also used to visualize and quantify lipid droplets, especially, droplets of neutral lipids within cells in oleaginous microorganisms such as *Candida* spp. [42,46]. Oleaginous yeasts can accumulate lipids in the range of 20 to 70% of their biomass under appropriate cultivation conditions [47]. Therefore, the reduced binding of Nile red to *C. albicans* cells does not indicate the only changes in membrane fluidity, but the significant reduction of the binding of both DPH and Nile red to myricetin-treated *C. albicans* cells suggests that myricetin induces changes in lipid components, such as ergosterol, phospholipids, or sphingolipids, in the cell membrane of myricetin-treated *C. albicans* cells. Thus, the enhanced membrane permeability and notable reduction of the entry of DPH into, and the binding of Nile red to, myricetin-treated *C. albicans* cells imply that myricetin increases membrane fluidity by inhibiting the biosynthetic pathways or functions of lipid components in the cell membrane, leading to perturbations in the structure and function of the cell membrane. To clarify this hypothesis, further studies will be needed. In conclusion, myricetin acts as an antifungal against *C. albicans* through a combined action of membrane disturbance by enhancing membrane permeability and cell wall damage. Thus, the presence of dual targets of myricetin against *C. albicans* indicates its potential as a therapeutic agent to treat infections caused by *Candida* spp.

## Figures and Tables

**Table 1:** MICs of myricetin against *Candida* spp.

	MIC (μg/ml)	

	Myricetin	Amphotericin B
*C. albicans* SC5314 (ATCC MYA-2876)	20	1
*C. glabrata* ATCC 2001 (KCCM 50044)	1.3	1
*C. krusei* ATCC 6258 (KCCM 11426)	5	0.5
*C. parapsilosis* ATCC 22019	5	1

The in vitro MICs of myricetin against *Candida* spp. were determined by the modified CLSI M27-A3 method containing resazurin.

**Table 2:** Sorbitol protection assay and MICs of myricetin against *C. albicans* SC5314.

	MIC (μg/ml)			

	24 h		72 h	

	RPMI	RPMI + sorbitol	RPMI	RPMI + sorbitol
Myricetin	20	40	20	80

Antifungal susceptibility tests were performed by the modified CLSI M27-A3 protocol containing resazurin without or with 0.8 M sorbitol, and MICs were determined after 24 and 72 h, respectively.

**Table 3:** Nile red-binding assay.

DMSO			Myricetin			*p*

Treatment (%)	Fluorescence (AU)	Relative fluorescence (%)	Treatment (µg/ml)	Fluorescence (AU)	Relative fluorescence (%)	
0.1	22671 ± 441	100.0 ± 1.9	20	16325 ± 865	72.0 ± 3.8	< 0.001
0.2	24916 ± 1009	100.0 ± 4.0	40	16097 ± 991	64.6 ± 4.0	< 0.001
0.4	27214 ± 1390	100.0 ± 5.1	80	15157 ± 481	55.7 ± 1.8	< 0.001

*C. albicans* cells treated with myricetin or an equivalent amount of DMSO were incubated in PBS containing 0.25 mg/ml Nile red. The amount of Nile red binding to *C. albicans* cells was measured at 488 nm (bandwidth, 20 nm) and 580 nm (bandwidth, 20 nm), as the excitation and emission wavelengths, respectively, using a spectrofluorometer. The data represent the means ± standard deviations obtained from one of three independent experiments. AU: arbitrary units.
